# Assessing the Effects of Ozonation on the Concentrations of Personal Care Products and Acute Toxicity in Sludges of Wastewater Treatment Plants

**DOI:** 10.3390/toxics11010075

**Published:** 2023-01-13

**Authors:** Chi-Ying Hsieh, Ya-Chin Wu, Sunaina Mudigonda, Hans-Uwe Dahms, Meng-Chun Wu

**Affiliations:** 1 Department of Environmental Science and Engineering, National Pingtung University of Science and Technology, Pingtung 912, Taiwan; 2 Center for Water Resources Education and Research, National Pingtung University of Science and Technology, Pingtung 912, Taiwan; 3 Department of Biomedical Science and Environmental Biology, Kaohsiung Medical University, Kaohsiung City 80708, Taiwan; 4 Department of Medicinal and Applied Chemistry, Kaohsiung Medical University, Kaohsiung City 807, Taiwan; 5 Department of Marine Biotechnology and Resources, National Sun Yat-sen University, Kaohsiung 80424, Taiwan; 6 Research Center for Precision Environmental Medicine, Kaohsiung Medical University, Kaohsiung 80708, Taiwan

**Keywords:** PCP remediation, wastewater treatment plants, nonylphenol, triclosan, benzophenone-3, caffeine, pressure-assisted ozonation, ecological risk, invertebrate model

## Abstract

The aim of this study was to understand the distribution of the personal care products nonylphenol (NP), triclosan (TCS), benzophenone-3 (BP-3), and caffeine in the sludges from three wastewater treatment plants (WWTP-A, -B, and -C) in southern Taiwan. The four compounds were analyzed from activated sludge and dewatered sludge samples, and then the samples were treated with pressure-assisted ozonation under different conditions and removal efficiencies. All four target compounds were detected, especially NP, which was detected in the highest concentrations in the activated sludges of WWTP-A and dewatered sludges of WWTP-C at 17.19 ± 4.10 and 2.41 ± 1.93 µg/g, respectively. TCS was dominant in dewatered sludges from WWTP-B, and the highest detected concentration was 13.29 ± 6.36 µg/g. Removals of 70% and 90% were attained under 150 psi at 40 cycles for NP and TCS, respectively, with 5 min of ozonation reaction time, a solid/water ratio of 1:20, and 2% ozone concentration. Ecological risk quotients (RQs) were calculated by the ratios of the 10-day *Hyalella azteca* (freshwater amphipod) LC_50_ to the environmental concentrations of the target compounds. High RQs were found to be >10 for NP, TCS, and BP-3 in untreated sludges, resulting in significant ecological risks to aquatic organisms when the sludges are arbitrarily disposed. However, the toxic effects on *Hyalella azteca* were not significantly different among ozone sludge treatments. The reason for this may be related to the formation of toxic oxidation by-products and incomplete mineralization of organic compounds. This could also be true for unknown intermediates. The relatively high detection frequencies of these emerging compounds in WWTP sludges requires further applications and treatments.

## 1. Introduction

Personal care products (PCPs) are among the main detected compounds in the environment, and some have been identified as endocrine disruptors [1,2,3]. The concentrations of these compounds, including nonylphenol (NP; an alkylphenol), triclosan (TCS; an antibacterial agent), benzophenone-3 (BP-3; a UV filter), and caffeine (CAF), are among the highest documented in wastewater treatment plants (WWTPs). However, the current treatment of organic pollutants, which mostly uses biological, chemical oxidation, and photochemical approaches, might not efficiently remove these compounds. The activated sludge process is an aerobic biological treatment of sewage. It is widely used for removing dissolved organic matter from wastewater [4]. Advanced oxidation processes (AOPs) were applied to treat organic compounds based on the formation of hydroxyl radicals and their quick, destructive reactions with water pollutants [5,6]. Nowadays, the application of pressure-assisted ozonation (PAO), a type of oxidation treatment, is used to accelerate the ozonation treatment of soil and sediment slurries contaminated by recalcitrant organics [7,8]. A pressure gradient (ΔP > 30 bar) was tested for sludge treatment to enhance floc disintegration and cell rupture, leading to increased volatile solid (VS) removal and biogas production [4,9,10]. Organic waterborne TCS and BPA disappear within minutes of pressure-assisted ozonation treatment from an initial amount of 9 mg/L [11]. Some PCPs have been shown to have adverse effects on endocrine systems and toxic effects on different organisms at low doses [12,13,14]. However, the different effects of such pollutants on the environment still need to be further clarified. *Hyalella azteca* was used as a toxicity test species for the evaluation of chemical toxicity from sediments and sludges. The US Environmental Protection Agency [15,16] recommended the amphipod *H. azteca* as a 10-day survival/lethality toxicity test in the standard sediment toxicity test protocol. The present study investigated four compounds (NP, TCS, BP-3, and CAF) from activated WWTP sludge and dewatered sludge samples that were further treated with pressure-assisted ozonation under different conditions. The use of the aquatic invertebrate *Hyalella azteca* as a toxicity testing species and the ecological risks of the target compounds in sludges and their toxicological effects are reported and evaluated herein.

## 2. Materials and Methods

### 2.1. Chemicals

Chemical standards used in this study were purchased in analytical grade. technical-nonylphenol (t-NP, 92.5%) and triclosan (TCS, 97%) were purchased from Fluka (Buchs, Switzerland), and benzophenone-3 (BP-3, >99.9%) was acquired from AccuStandard (New Haven, CT, USA). Anhydrous caffeine powder (99%) was supplied by Alfa Aesar (Karlsruhe, Germany). All organic solvents used in the following sample processing and analysis were HPLC grade and obtained from Merck Corporation (Darmstadt, Germany) and/or Echo Chemical (Miaoli, Taiwan). HPLC- grade water was obtained from a Milli-Q water purification system (Millipore, Watford, USA). Oasis HLB cartridges (500 mg, 6 mL) were purchased from Waters Corporation (Milford, MA, USA). A 100 μg/mL stock solution was prepared in MeOH and stored in amber glass vials. The stock solutions of standards were then diluted to the appropriate concentrations with MeOH to serve as working solutions. All stock and working solutions were stored at −20 °C in the dark.

### 2.2. Sampling

In the present study, six activated and dewatered sludge samples were collected from three wastewater treatment plants (code-named WWTP-A, WWTP-B, and WWTP-C) located in southern Taiwan. The relative locations of the sampling sites in the three WWTPs are shown in Figure S1. WWTP-A has a site area of 9.99 ha, and the wastewater source for treatment is mainly domestic sewage. At present, the actual treatment capacity is about 20,000 CMD; the biological treatment unit is the A2O system (anaerobic–anoxic–oxic process), and then sodium hypochlorite is used for disinfection procedures, and the hydraulic retention time (HRT) is designated as 9 days. WWTP-B has a site area of 10 ha. Its water source is mainly domestic sewage; its treatment capacity is 31,000 CMD. Disinfection procedures are carried out with sodium hypochlorite, and HRT is designated as 7 to 8 h. WWTP-C has a site area of 2.95 ha. The main source of treated water is intercepted stream water (accounting for about 2/3), followed by domestic sewage. The average sewage treatment capacity is only 2979 CMD. The biological treatment unit allows extended aeration followed by UV disinfection before discharge. The hydraulic retention time (HRT) is designated as 1.5 days.

### 2.3. Sample Preparation

A freeze dryer was used to remove moisture content from the collected sludge, which was sieved (particle size < 150 μm) and extracted in a methanol–acetone solution (1:1, *v*/*v*) by ultrasonication. The slurry was centrifuged, concentrated with a rotary evaporator, and reconstituted with dichloromethane/methanol. Extracts were purged with nitrogen at 35 °C until dry and reconstituted with 50% ACN/water. Final solutions were filtered through 0.22 µm PVDF filters and transferred into amber vials to prevent photo-degradation of selected analytes until further analysis was performed with high-performance liquid chromatography with UV−Vis detection (HPLC-UV) and fluorescence detection (HPLC-FLD).

### 2.4. Ozone Treatment Procedure

**Figure 1:**
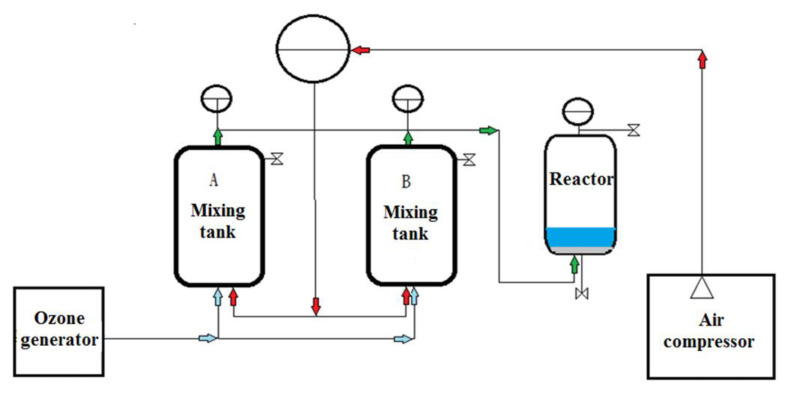
Scheme of pressure-assisted oxidation system (PAO).

The experimental system for the treatment of target PCPs by pressure-assisted ozonation (PAO) is shown in Figure 1. Briefly, an ozone/air mixture was compressed by an air compressor and introduced into a reactor with a closed headspace above the treated contaminated water. Compression was carried out to a prescribed elevated pressure in the headspace, thus oversaturating the water with air and ozone beyond what is normal at ambient pressure. The optimized experimental operating parameters for the sludge/H_2_O ratios, compression and decompression cycles, ozone concentration (%), contact time (min), and pressure (psi) were at 1:20 (minimum), 40 cycles, 2% ozone, 5 min, and 150 psi, respectively.

### 2.5. Apparatus and Chromatographic Conditions

The HPLC system (Waters, 2695 model), coupled with a UV−Vis photodiode array detector (Waters, 2996 model) and a fluorescence detector (Waters, 2475 model), was used for analysis and quantification of target compounds. The data processing was carried out with Empower software (Waters Co., Milford, MA, USA). The analytical column used for the target compound was Waters RP18 (4.6 × 250 mm, 5 μm), and the flow rate of the mobile phase was set at 1 mL/min. The mobile phase contains ultrapure water (B) and acetonitrile (C). The elution condition is that the mobile phase is eluted from a gradient of 30:70 → 0:100 → 30:70 within 20 min to separate the target compound for detection. Compounds TCS, BP-3 and CAF were detected by UV and NP were detected by a fluorescence detector., and the sample injection volume was 20 μL. In addition, the mobile phase containing 0.5% TFA in ultrapure water (A) and acetonitrile (C) was used to detect caffeine, and the sample injection volume was 20 μL. The instrument settings and detection conditions for each compound are shown in Table S1.

### 2.6. Biological Toxicity Test

A whole-sludge sample toxicity test was carried out using the standard toxicity test invertebrate amphipod *Hyalella azteca*. The US EPA [17], ASTM (2010) [16], and Taiwan EPA methods (2016) were followed to conduct a 10-day survival test.

In this study, different concentrations of NP, TCS, BP-3, and caffeine were spiked into reference sediments according to EPA Canada (2013) [18]. Whole-sludge samples were tested for toxicity using a 10-day *Hyalella azteca* toxicity test. When the sludge concentration was stable, the actual concentration in the spiked sludge was confirmed, and *H. azteca* was exposed to different concentrations to determine the dose–response relationship as well as obtain the LC50 values of NP, TCS, BP-3, and caffeine. Then, RQ was used to estimate the risk of the target compound in the benthic ecological environment. EEC is the actual detection of the environmental samples’ highest concentration value.

In order to discuss whether the biological survival rate can be improved by PAO treatment, the activated and dewatered raw sludge samples were collected from the three WWTPs after the PAO treatment of the samples. In addition, in order to better understand the relationship between pollutants and amphipods and calculate the ecological risks, the concept of adding chemical substances to the reference sediment (sediment spiking) was applied to determine the laboratory toxicity of specific pollutants, and a dose–response curve was established using different concentrations to obtain the LC_50_ of NP, TCS, BP-3, and caffeine.

### 2.7. Quality Assurance and Quality Control for Chemical Analysis 

A quality control sample (100 μg/L standard) was run after every tenth injection, followed by a blank sample (ACN/water). Calibration curves for the analyte were developed in the range of 0.1–12 mg/L, and the determination coefficients (R2) obtained were >0.995 for all the compounds (shown in Table S2). 

For the recovery rate test, a standard of known concentration was added to the blank soil sample (a blank sludge sample was prepared in equal proportions of clay and dolomite), treated by the steps of sludge pre-treatment, and finally returned to volume with acetonitrile/ultrapure water, filtered through a 0.22 µm filter head, and placed in a brown glass vial using HPLC- UV-FLD for analysis. Recoveries were typically above 70% in this study. 

### 2.8. Biological Toxicity Test

The QA/QC of the biological test followed the quality control requirements of the American Society for Testing and Materials (ASTM E1706-05), including performing duplicate quality control analysis; ensuring a survival rate of the control group greater than 80%; performing an organism health test with standard sodium chloride (NaCl); preparing the reference sediment with white quartz sand, cellulose, silt, clay, dolomite CaMg (CO_3_)_2_, and humic acid providing appropriate organic carbon source for test organisms as a test system control group; and monitoring the quality of the overlying water during the test.

### 2.9. Ecotoxicity Risk Assessment

Ecological risk assessment was performed based on the RQ index in line with the established protocol [19,20,21,22]. The RQ evaluation was derived from the integration of using the Environmental Fate and Effects Division (EFED) as the results of the exposure and ecotoxicity data. RQs were calculated by dividing exposure estimates by the acute and chronic ecotoxicity values (i.e., RQ = Exposure/toxicity). In accordance with the US EPA, the RQs for acute toxicity of aquatic invertebrates were calculated based on LC_50_ or EC_50_. 

## 3. Results and Discussion

### 3.1. Concentrations of Target Compounds in Wastewater Treatment Plant Sludges

**Figure 2:**
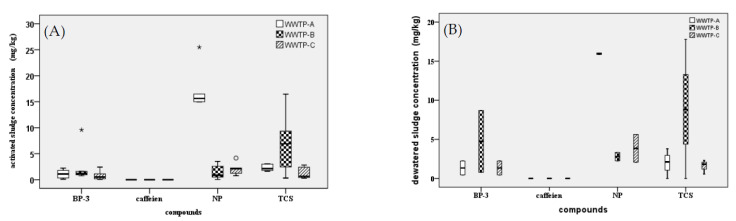
Target compounds in activated (**A**) and dewatered (**B**) sludges from three WWTPs. O represent outliers; * represent extreme values.

The distribution of the four target compounds in activated and dewatered sludges from the three WWTPs is shown in Figure 2. Overall, NP, TCS, and BP-3 were detected in all sludge samples from the WWTPs. NP concentrations were highest in the activated sludge of WWTP-A and dewatered sludge of WWTP-C at 17.19 ± 4.10 and 2.41 ± 1.93 µg/g, respectively. TCS was dominant in dewatered sludge from WWTP-B and WWTP-C, the highest concentrations detected being 13.29 ± 6.36 µg/g and 4.74 ± 5.59 µg/g, respectively. According to a Canadian research survey, the NP concentrations ranged from 4.6 to 1230 µg/g (with a median concentration of 232 µg/g in 35 sewage sludge samples); TCS had a concentration range from 0.90 to 28.2 µg/g and a median of 12.5 µg/g [23], which showed that, compared with other countries, the average concentration of NP in this study was lower, while the concentration of TCS was similar. The elevated NP levels are likely due to the fact that the sewage treatment plants of those cities are receiving inputs from nearby textile industries [24]. Our study showed that the concentrations of BP-3 in the activated and dewatered sludge of the three WWTPs were 0.79 ± 0.89~2.54 ± 3.46 µg/g and 0.46 ± 0.52~4.74 ± 5.59 µg/g, respectively; the concentration of BP-3 in WWTP-A was the highest and that in WWTP-C was the lowest. The concentrations of BP-3 in the sludges of the present study were similar to those reported in Spain (0.79 μg/g dw) [25] and southern Australia (0.149 ± 0.013 and 0.303 ± 0.026) [26], but higher than those determined in the Xiamen (BLD~0.0275 μg/g) [27], Hunan (1.94 ng/g) [28], and Guangzhou provinces of China (11.9 ± 1.77 and 36.7 ± 7.49 ng/g) [29]. Caffeine was not detected in the WWTP sludges and should be related to the adsorption coefficient (Log Kow = −1); it is, therefore, not easily adsorbed by solid sludge. The high concentrations of NP, TCS, and BP-3 indicated that the tested wastewaters were predominantly from municipal sources. 

### 3.2. Removal Efficiencies in WWTP Processes

**Figure 3:**
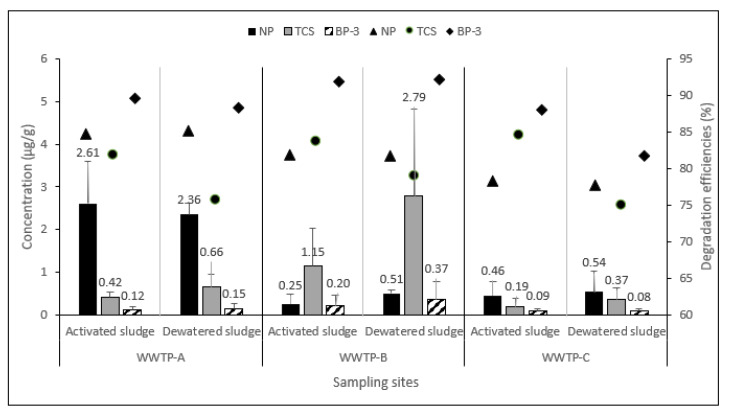
Comparison of three compound concentrations and degradation efficiencies (NP: nonylphenol; TCS: triclosan; BP-3: benzophenone-3) after treatment with PAO in three WWTPs. ■ Concentration of compounds (error bars represent standard deviation of the mean value, *n* = 6); ▲ degradation efficiencies of NP; ● degradation efficiencies of TCS; ◆ degradation efficiencies of BP-3.

The concentrations of NP, TCS, and BP3 in activated and dewatered sludges after PAO treatment are shown in Figure 3. NP is a widely detected compound and our study showed that NP was still detectable in WWTP-A at concentrations up to eight times higher than at the other two WWTPs. The concentrations of NP in raw sludge ranged from 1.4 to 17.2 µg/g. Our results indicate that the removal efficiencies of all compounds were up to 70% even though sludges are complex mixtures, and the removal efficiencies by PAO treatments in the current research were similar to those reported in the literature. This is slightly lower than the maximal 95% removal efficiency of ozone and chlorine disinfection reported in WWTPs [30]. Our results showed that TCS concentrations varied from 1.2 to 13.29 µg/g in the three WWTPs, with the highest concentrations detected in the raw sludge of WWTP-B. The ozone process is capable of depleting TCS effectively [31], and our experimental results showed that TCS concentrations were 0.2–2.8 µg/g and removal efficiencies were up to 80% for the PAO treatment in all WWTPs. That TCS and BPA were readily degradable by ozonation under PAO conditions indicated that TCS and BPA can be removed to a percentage greater than 98% for only 6 min under a lower pressure and lower ozone concentration [12]. In the present study, BP-3 concentrations were detected at 0.5~4.7 µg/g in all raw sludges, and other studies demonstrated sunscreen (BP-3) removal at 20–90% using ozone [32]. The highest concentration was detected in the raw sludge of WWTP-B. Our experimental results with PAO treatment resulted in BP-3 concentrations of 0.1–0.4 µg/g and removal efficiencies of approximately 90%, similar to results reported in the literature [33,34].

Caffeine levels in digestion and dewatering sludges were all below the detection limit. Its log K_ow_ value was around -0.07 [35] and it was not substantially adsorbed to the solid phase. However, ozone can remove >80% of caffeine [36]. Overall, the degradation efficiencies of NP and TCS were up to 70% and 90%, respectively, under the experimental conditions of our study.

### 3.3. Sludge Toxicity Test with Hyalella Azteca 

**Figure 4:**
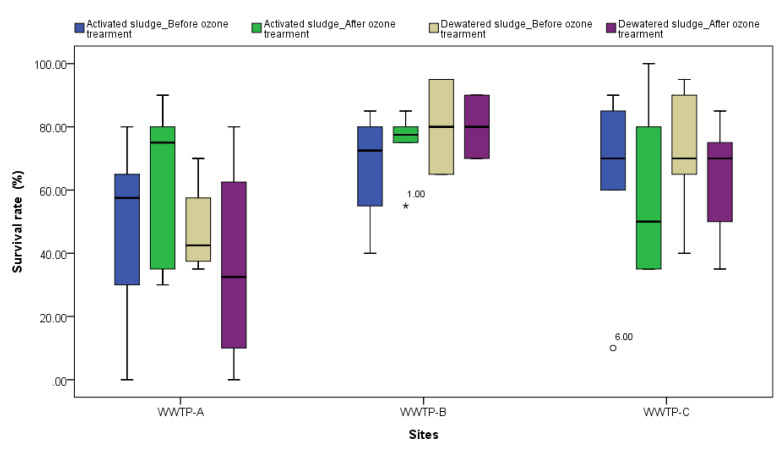
Survival rates of *H. azteca* exposed to sludges from three WWTPs.

A 10-day survival/mortality toxicity test with *Hyalella azteca* was used to assess sludge toxicity according to Environment Canada protocols [31,32]. The mean survival rate of the control amphipods was >80%, which met the protocol criterion. The *H. azteca* survival rate of WWTP-A and WWTP-B activated sludge before and after PAO treatment was 48.33 ± 29.10% and 67.50 ± 17.54%, respectively, which increased to 64.17 ± 25.38% and 75.00 ± 10.49%, while the survival rate of WWTP-C decreased slightly (64.17 ± 28.71 → 58.33 ± 26.39). For the dewatered sludge, the survival rate of WWTP-B was almost the same before and after PAO treatment, but the survival rate of WWTP-A and WWTP-C decreased slightly by about 10% after PAO treatment. The results of one-way ANOVA and LSD multiple comparison test showed that there was no significant difference (*p* < 0.05) in any of the samples before and after PAO treatment. The survival rates of *H. azteca* in whole sludge before and after PAO treatment are provided in Figure 4. Eight PAO-treated samples had higher survival rates. There were no significant correlations between survival and the concentrations of various pollutants (data not shown). The residual ozone in the reactions was assumed to have small effects on sludges as other studies indicated that only 1% of the ozone (at 20 ℃) remained after 20 min [37]. However, the physical and chemical properties of sludges and compounds or oxidation by-products will change the survival rate, dependent on PAO parameters. Another study also indicated that TCS can transform through methylation into methyl triclosan, which is relatively stable under photodegradation exposure and can accumulate in organisms [38]. Ozonation carries the inherent danger of producing toxic oxidation by-products because chemical compounds are often not mineralized entirely but transformed into unknown intermediates instead [36,39]. It has been reported that adverse effects occurring after the ozone reactor are possibly due to the formation of toxic oxidation by-products [40,41]. Since there was no significant correlation when using *H. azteca* toxicity to test ozonation in sludge before and after treatment, chronic toxicity tests can be used to evaluate growth/reproductive effects in the future.

### 3.4. Ecological Risk Quotient

A 10-day *H. azteca* toxicity test was used to determine ecological risk. The 50% lethal concentration (LC_50_) tests conducted for NP, TCS, and BP-3 resulted in values of 0.4000, 0.4777, and 0.7721 µg/g, respectively. The risk quotient (RQ) approach, calculated as the ratio of estimated or measured environmental concentrations (EEC) to toxicity test effect levels (LC_50_, LD_50_, or NOAEC), was then used to characterize the eco-toxicological risks posed by NP, TCS, and BP-3 based on US EPA guidelines.

**Figure 5:**
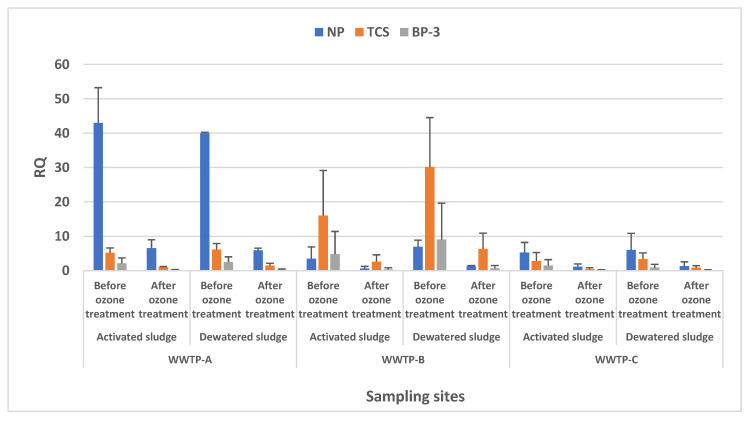
Calculated risk quotients of NP, TCS, and BP-3 of different sludges.

RQ values are shown in Figure 5 as well as the accepted ranges for RQ values, where low risk was <0.1, medium risk was 0.1–1.0, and high risk was >10. In WWTP-A, the RQ values for NP, TCS, and BP3 decreased from 64 to 10.9, 8.6 to 2.5, and 4.2 to 0.5, respectively. The risks generally dropped for the three compounds. However, NP still posed a higher risk compared with BP-3.

In WWTP-B, the calculated RQs for NP, TCS, and BP-3 decreased from 9 to 1.5, 40 to 9.6, and 18 to 1.3, respectively, indicating that high risks dropped to moderate risks. In WWTP-C, the RQ values for NP, TCS, and BP-3 decreased from 14 to 3.4, 6.4 to 1.3, and 4.6 to 0.3, respectively. At the same time, environmental risks decreased from moderate to low. RQ calculations proved that post-treatment with ozone can indeed reduce the biological effects of personal care products in sludge, although the calculated risk quotient of BP-3 was less than that of NP and TCS in WWTP-C. However, the large quantities and widespread use of sunscreens indicated that intense monitoring is necessary [11,42]. During six sampling analyses, caffeine did not adsorb appreciably to sludges (log Kow = −0.07) but was among the most frequently detected compounds. Previously published reports indicated that high concentrations of caffeine affected the development of *Xenopus laevis* eggs and increased the teratogenesis of embryos [43].

## 4. Conclusions

Our results showed that, among the three WWTPs, the detection rates of the target compounds (NP, TCS, and BP-3) were up to 100%. The activated sludge sample detection rate of NP was highest in WWTP-A, with concentrations up to 17.19 ± 4.10 µg/g. The dewatered sludge in WWTP-B had an average TCS concentration of 13.29 ± 6.36 µg/g, and the highest detectable NP concentration in WWTP-C dewatered sludge was 2.41 ± 1.93 µg/g. The removal efficiencies of NP, TCS, and BP-3 were up to 70%, 80%, and 90% by pressure-assisted oxidation, respectively. The calculated RQ was >10 for some target compounds in sludges without treatment, which posed greater ecological risk to aquatic organisms. Although there were no significant differences when using *H. azteca* to test the toxicities of the sludges before and after ozonation treatment, the ecological risk assessment using a single compound was often found to be overrated. Therefore, whole-organism tests conducted on-site in flow-through systems would be important for evaluating the toxicity of WWTPs after ozonation as well as the detoxification potential of post-treatments because substance loss was minimized. Therefore, we propose to conduct further chronic toxicity tests.

## Data Availability

Not applicable.

## Supplementary Materials

The following supporting information can be downloaded at: https://www.mdpi.com/article/10.3390/toxics11010075/s1, Figure S1: Location map of the study area in Taiwan; Table S1: Instrument parameter conditions of the target compound; Table S2: Validation parameters of the method for quantitative analysis of target compound.
