# Supplementary material for: Assessing the Effects of Ozonation on the Concentrations of Personal Care Products and Acute Toxicity in Sludges of Wastewater Treatment Plants
Source: Toxics. 2023 Jan 13;11(1):75. doi: 10.3390/toxics11010075 (PMC9865304; doi:10.3390/toxics11010075)
Supplement: Supplementary file 1 [file toxics-11-00075-s001.zip › toxics-2064442-supplementary.pdf]

Supplementary Materials

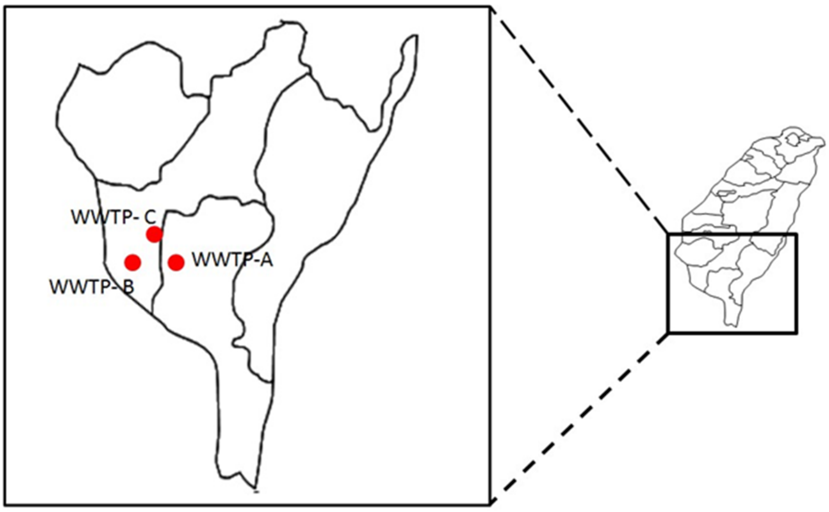

Figure S1. Location map of the study area in Taiwan.

Table S1. Instrument parameter conditions of the target compound

Compound	Retention time (min)	UV (nm)	Excitation	Emission
NP	9.2	-	227	313
TCS	7.8	220	-	-
BP-3	5.4	220	-	-
CAF	6.8	273	-	-

Table S2. Validation parameters of the method for quantitative analysis of target compound

Compound	Linearity		Recovery (%)	MDL (ng/L)	LOQ (ng/L)
	Equation	Correlation Coefficient (R)			
NP	y = 40.486x - 1620.4	0.9998	110 ± 6.09	15	45
TCS	y = 69.115x + 1535.7	0.9999	92 ± 1.85	15	45
BP-3	y = 62.558 + 1062.9	0.9999	80 ± 2.59	20	60

CAF	y = 26.564x - 7271	0.9951	110 ± 6.94	25	75
